# Central venous catheter care and the healthcare–environment interface: outcomes of a quality improvement initiative in a resource-constrained ICU

**DOI:** 10.3389/fpubh.2025.1713370

**Published:** 2025-12-12

**Authors:** Rehab H. El-Sokkary, Essamedin M. Negm, Fayrouz Abd ElNaser, Al Zahraa Mohammed Soliman, Amira Hamed Mohamed Afifi, Samar S. A. Morsi, Alaa O. Abdel-Kareem, Afaf M. Eladl, Mona H. Ibrahim, Maha Abdeldayem, Mai M. Malek

**Affiliations:** 1 Medical Microbiology and Immunology, Faculty of Medicine, Zagazig University, Zagazig, Egypt; 2 Anesthesia, Intensive Care, Pain management Department, Faculty of Medicine, Zagazig University, Zagazig, Egypt; 3 Clinical Pharmacist in Emergency Surgical ICU at Zagazig University Hospitals, Zagazig, Egypt; 4 Public Health and Community Medicine, Faculty of Medicine, Zagazig University, Zagazig, Egypt; 5 Clinical Pathology, Faculty of Medicine, Zagazig University, Zagazig, Egypt

**Keywords:** bundle, CLABSI, quality improvement, CVC maintenance, compliance, sustainability in healthcare

## Abstract

**Background:**

Central line-associated bloodstream infections (CLABSI) are major indicators of healthcare quality and patient safety, particularly in resource-constrained intensive care units (ICUs).

**Aim:**

This study aimed to evaluate a pre-post quality improvement (QI) initiative designed to optimize central venous catheter (CVC) insertion and maintenance, reducing CLABSI rates, and promoting more sustainable critical care practices in a university hospital ICU.

**Methodology:**

A one-year pre–post QI study (October 2022–September 2023) was conducted in an emergency surgical ICU of a university hospital in Egypt hospital. The study was structured according to the Standards for Quality Improvement Reporting Excellence (SQUIRE 2.0) framework and using sequential Plan-Do-Study-Act (PDSA) cycles. All eligible ICU patients needing CVCs were included. The intervention included: (1) standardizing CLABSI definitions and rate calculations, (2) introducing CVC insertion and maintenance checklists with daily audits, (3) targeted staff education and training, and (4) forming a multidisciplinary CLABSI working group for monitoring and feedback. Over 6 months, process improvements were implemented, followed by 3 months of impact measurement, including assessing CVC insertion/maintenance compliance, CLABSI rates, and device utilization ratio (DUR), with all data collected manually.

**Results:**

A total of 1,370 patients and 2,277 CVC days were observed. The CLABSI rate declined from 7.56 ± 2.26 to 6.97 ± 1.31 per 1,000 CVC days (−13.4%), and DUR decreased significantly from 2.21 ± 0.34 to 0.98 ± 0.19 (*p* = 0.02). Maintenance bundle compliance improved from 39.5 to 59.7% (*p* = 0.01), and insertion compliance increased from 62.5 to 72% (*p* = 0.6). Hand hygiene adherence demonstrated a strong negative correlation with CLABSI rates (*r* = −0.95, *p* = 0.02).

**Conclusion:**

This single-center study had a small sample size and short post-intervention follow-up, which may limit generalizability. Nonetheless, the initiative demonstrates that structured, low-cost QI interventions can improve compliance, reduce device use, and may support safer, more environmentally sustainable ICU care. Continuous monitoring and multicenter validation that integrate infection control with environmental stewardship are essential to sustain improvements and minimize the environmental footprint of critical care. Digital surveillance and ongoing training can improve real-time monitoring and scalability in healthcare systems.

## Background

The central venous catheter (CVC) is an intravascular line that terminates near the heart or within one of the major vessels. It is used for fluid infusion, blood withdrawal, and hemodynamic monitoring (1). Its use increases the risk of potentially fatal bloodstream infections (2). Many health care institutions have set targets to minimize central line-associated bloodstream infections (CLABSIs) to enhance patient outcomes and lower healthcare expenses (3).

Another term that is frequently used by clinicians, catheter-related bloodstream infection (CRBSI) necessitates particular test evidence that the infection originated in the catheter. Usually, differential time-to-positivity testing or quantitative blood cultures are used for this—which are not always feasible in routine clinical practice. The Centers for Disease Control and Prevention (CDC) employs a more straightforward surveillance definition, known as CLABSI, which is a primary bloodstream infection that occurs in a patient who had a central line within 48 h prior to infection onset and has no other identifiable source.

Approximately 250,000 bloodstream infections occur annually in United States hospitals, with nearly 80,000 CLABSIs reported in intensive care units (ICUs) each year (4). The International Nosocomial Infection Control Consortium (INICC) estimates a pooled incidence of 4.1 per 1,000 central line-days over 5 years (5). Middle-income countries report considerably higher CLABSI rates than high-income countries, likely reflecting disparities in infrastructure, resources, and adherence to prevention protocols. In Egypt, for example, a multicenter study across three ICUs reported an average CLABSI rate of 9.1 per 1,000 central line-days (6).

Multiple factors contribute to elevated CLABSI rates, including inconsistent aseptic technique during insertion or maintenance, lack of standardized surveillance definitions, poor documentation, and communication gaps within healthcare teams (6–9). These challenges not only compromise patient safety but also increase antibiotic consumption, length of stay, and medical waste, creating additional strain on already limited resources.

To address this, evidence-based prevention bundles have been promoted globally. The Institute for Healthcare Improvement (IHI) introduced the “bundle” concept in 2001 as part of its collaboration with the Voluntary Hospital Association (VHA), leading to significant reductions in CLABSI rates across multiple settings (10–12). However, sustained success depends on cultural change, consistent adherence, and continuous quality monitoring—particularly challenging in low-resource environments.

Quality improvement strategies offer a systematic approach to modifying processes and improving outcomes in a sustainable manner (9, 13, 14). By optimizing CVC insertion and maintenance, these initiatives can improve patient safety, conserve resources, and reduce the environmental footprint of healthcare delivery (15). In low middle income countries (LMICs), evidence-based prevention bundles, CVC care guidelines, and some training programs are available and can be adapted to local settings. However, consistent access to trained personnel, adequate surveillance infrastructure, resources for sustained quality improvement, and full integration of infection prevention into routine ICU practice remain limited. These gaps make maintaining long-term reductions in CLABSI rates particularly challenging in low-resource environments (9, 13, 15).

This study therefore aimed to evaluate a pre-post QI initiative designed to optimize CVC insertion and maintenance, reducing CLABSI rates, and promoting more sustainable critical care practices in a university hospital ICU.

## Methods

This pre-post quality improvement study using multiple Plan-Do-Study-Act (PDSA) cycles was conducted from October 2022 to September 2023. To ensure the transparency and the completeness in reporting, this study was structured in accordance with the Standards for Quality Improvement Reporting Excellence (SQUIRE 2.0) guidelines, as recommended by the Enhancing the Quality and Transparency Of health Research (EQUATOR) Network (A Supplementary file) into: Context, Intervention, Study of the Intervention, Measures, Analysis, and Results.

### Context

This initiative was conducted in the emergency surgical ICU of a university hospital in Egypt, a resource-constrained setting with a high burden of CLABSIs to identify causes of elevated CLABSI rates and select interventions. Before the intervention, the unit lacked standardized CLABSI definitions, updated CVC care policies, and structured staff training. Frequent turnover of junior staff, absence of insertion qualifications, and limited infection control oversight further contributed to infection risk. These contextual factors shaped the design and implementation of the interventions and highlighted the necessity of tailoring improvement strategies to local conditions.

The Quality Improvement approach was chosen for its capacity to systematically identify gaps in care, implement targeted, evidence-based intervention, and facilitate ongoing monitoring through iterative cycles. This methodology enables timely adjustments, fosters staff involvement, and promotes adherence to best practices within high-risk and dynamic settings.

#### Sample size and type

Accessibility sampling method in which all patients admitted to the investigated unit, met the inclusion criteria and had complete medical records were included in the study. Inclusion criteria comprised all patients admitted to the emergency surgical ICU who met the following: no documented infection at the time of admission, insertion of a CVC during their ICU stay, central line should be in place two calendar days and development of CLABSI without evidence of a remote source of infection (1). Exclusion criteria included intubation on admission, presence of central or urinary catheters before admission, recent hospitalization (within 14 days), immunosuppression, or chronic illnesses such as end-stage renal disease or decompensated liver cirrhosis (7). These exclusions aimed to reduce confounding from non-CVC-related infections (e.g., Ventilator associated pneumonia) and underlying conditions that independently increase infection risk (e.g., chronic diseases), thereby preserving the internal validity of the intervention’s impact.

#### Pre-intervention

During this 3-months phase, we measured CLABSI rate, audited the CVC insertion and maintenance processes, interviewed various stakeholders, and reviewed available records. Utilizing QI tools such as a process map, fishbone diagram, and driver diagram, we identified factors that likely contributed to the increased CLABSI rate.

### Intervention

**Figure 1:**
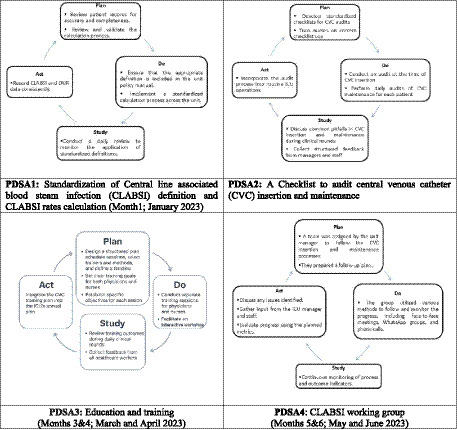
The six-month intervention was structured around four sequential PDSA cycles. In Month 1, PDSA 1 focused on standardizing the CLABSI definition and calculation method. Month 2 involved PDSA 2, which introduced checklists to audit CVC insertion and maintenance. Months 3 and 4 were dedicated to PDSA 3, delivering targeted education and training sessions for nurses and physicians. Finally, Months 5 and 6 implemented PDSA 4, establishing a multidisciplinary CLABSI working group to sustain and monitor the intervention efforts.

The intervention phase spanned 6 months and involved four PDSA cycles (Figure 1). Interventions were based on a prior unpublished QI study and refined iteratively during implementation. It was carried out by a multidisciplinary team, with specific roles assigned to nurses, physicians, and infection control professionals.

#### PDSA1 (first month of intervention); standardization of CLABSI definition and calculation of CLABSI rates

Prior to the intervention, inconsistent definitions and documentation practices hindered accurate tracking of CLABSI rates. In this context, standardizing the definition and method of calculation were a crucial first step to improve data reliability and provide a foundation for assessing the impact of later interventions. It was led by the infection prevention and control (IPC) team in collaboration with ICU physicians, who reviewed definitions and ensured alignment with CDC guidelines. Optimizing this step enabled the team to monitor the process of CVC insertion and maintenance in PDSA2. Discrepancies in documentation and variation in interpretation highlighted the need for training in PDSA3.

#### PDSA 2 (second month of intervention); introduction of CVC insertion and maintenance checklists with daily audits

Structured monitoring tools for CVC insertion and maintenance practices were introduced (16, 17). Clinical audits of physicians’ and nurses’ practices related to CVC insertion and maintenance were conducted daily using purpose-designed audit tools. An independent audit process was applied by an assigned team comprising members from the IPC unit and a clinical pharmacist. This helped minimize potential bias from peer observation. While inter-rater reliability was not formally measured, consistency was supported through auditor training and oversight by senior IPC personnel to ensure standardized checklist use and data accuracy.

Checklist audits revealed recurring errors and gaps in practice, especially among junior staff. These findings reinforced the need for targeted education addressed in PDSA 3.

#### PDSA 3 (third, and fourth months of intervention); education and training programs

Audit data and clinical observations revealed variation in technique and knowledge gaps. Two tailored educational programs were developed, one for nurses and one for physicians. The physician education program was conducted by Clinical educators and senior ICU physicians through interactive workshops, didactic sessions, and discussions during daily clinical rounds. The program aimed to strengthen skills in proper CVC insertion and deepen understanding of CLABSI management. The nursing program focused on enhancing infection prevention practices related to CVC insertion and maintenance, The IPC team delivered hands-on training and instructional videos for nurses.

Post-training audits showed improved compliance but also identified a need for ongoing support and accountability, which informed the creation of the CLABSI working group in PDSA 4.

#### PDSA 4(fifth, sixth months of intervention); formation of a CLABSI working group

To sustain improvements and address observed challenges in consistency, a multidisciplinary CLABSI working group was formed. This team, led by the unit manager and including infection control staff, monitored adherence, supported staff, and reinforced standards. The working group served as a feedback loop for real-time problem-solving and allowed for adaptive reinforcement of practices introduced in earlier cycles.

### Study of the intervention

The study assessed the intervention’s impact using pre-, during-, and post-intervention comparisons over a one-year period (October 2022–September 2023). Baseline data and stakeholder input identified key gaps in CVC insertion and maintenance. Process and outcome indicators were monitored throughout to evaluate changes attributable to the interventions. Trends and correlations were explored to understand intervention effects and their sustainability.

#### Measures

I Process indicators: They were used to track the execution of preventative measures of CVC insertion and maintenance. The central line insertion and maintenance bundle was explicitly mapped to international evidence-based guidelines, including the CDC, SHEA/IDSA, and world health organization (WHO) CLABSI prevention recommendations (2, 3, 11, 18). This mapping ensured alignment with globally accepted standards and strengthened the scientific rigor and generalizability of the intervention to similar low-resource ICUs (16, 17, 19, 20) as shown in Table 1. It is important to note that line lock or block solutions (such as heparin, ethanol, EDTA, taurolidine, or citrate) were not part of the implemented maintenance bundle. In our setting, CVC patency was maintained using normal saline flushes according to the institutional protocol.

(a) CVC insertion compliance rate: We calculated full compliance by dividing the number of compliant insertions by the total insertions and multiplying by 100. Data was collected using nurse-completed checklists (19). Full compliance required full adherence to all of the following: hand hygiene, appropriate skin preparation (Chlorhexidine gluconate for patients ≥60 days old or alternatives for younger children), allowing the antiseptic to dry before insertion, and use of all five maximal sterile barriers—sterile gloves, gown, cap, mask, and a large sterile drape (1).
(b) CVC maintenance compliance rates: We measured adherence to all bundle components. Full compliance was defined as a “yes” to all bundle items and compliance rate was calculated by dividing the number of fully compliant cases by the total number of CVC maintenance cases, then multiplying by 100 (17). Nurses collected daily audit sheets for each patient with an inserted CVC. For selected checklist items, the adherence rate to each selected item was calculated by dividing the number of compliant practices to this item by the total number of cared-for CVC cases and then multiplying by 100 (17).

II Outcome indicators:

(a) CLABSI Rate: The number of CLABSIs divided by number of CVC days multiplied by 1,000 (1).
(b) Device utilization ratio (DUR): Ratio of CVC days to patient days (21). Data were manually collected from patients’ records and checklists by assigned team members.

**Table 1:** Alignment of CLABSI bundle components with international guidelines.

Bundle component	CDC (2022)	SHEA/IDSA (2014 update)	WHO (2016)	Notes/local adaptation
Hand hygiene before catheter insertion	Recommended (Category IA)	Strong recommendation	Core IPC component	Alcohol-based hand rub used; periodic staff competency audits conducted
Maximal sterile barrier precautions during insertion	Recommended (Category IA)	Strong recommendation	Endorsed	Full barrier maintained; sterile drape ensured
Chlorhexidine-based skin antisepsis	2% CHG in alcohol preferred (Category IA)	Strong recommendation	Endorsed	CHG used when available; povidone-iodine substituted during shortage
Optimal catheter site selection (avoid femoral)	Recommended (Category IA)	Strong recommendation	Consistent	Internal jugular/subclavian preferred unless contraindicated
Daily assessment of line necessity	Recommended	Strong recommendation	Consistent	Daily ICU checklist used
Catheter hub/connector disinfection	Recommended	Strong recommendation	Endorsed	“Scrub-the-hub” ≥ 15 s protocol implemented
Closed/needleless systems	Recommended	Endorsed	Endorsed	Adopted according to availability
Transparent dressing and change policy	Recommended	Strong recommendation	Consistent	Dressing replaced every 7 days or when soiled
Ongoing staff education and compliance monitoring	Recommended	Strong recommendation	Core IPC component	education sessions and audit-feedback cycles maintained

### Analysis

Data analysis was performed using the SPSS (Statistical Package for the Social Sciences, version 25) (22). Cases with missing data (<5%) were excluded from the relevant analyses using list wise deletion method, as the missingness pattern was random and minimal, in which entire rows (cases) that have a missing value on any of the variables included in the analysis were removed. When appropriate, quantitative data were presented as mean and standard deviations or median and interquartile range (IQR). For quantitative non-parametric data, independent Kruskal Wallis test was used to compare CLABSI rate & DUR between the three phases, Post-Hoc Bonferroni test was used to find the difference within the three phases. Wilcoxon signed rank test and paired t-test were used for comparing insertion and maintenance compliance rates between the intervention and postintervention phases respectively, Spearman correlation analysis was done to find the association between CLABSI rate with insertion and maintenance compliance bundles. 80.0% test power was used, and 95% CI was calculated. *p* < 0.05 was considered statistically significant. While percentage changes in CLABSI rates are presented to illustrate trends, these were interpreted with caution due to the small number of events in each phase.

## Results

The study was conducted in three phases and included 1,370 patients aged 19–78 years (approximately 60% male and 40% female). Participants had comparable clinical characteristics, including Glasgow Coma scores at admission, past medical history, indication of hospitalization and ICU admission, lab investigation at admission, APATCHI II score, SOFA score, with no statistically significant differences among phases. Most patients were admitted to the investigated unit due to shock or respiratory failure or as a perioperative admission.

During the pre-intervention phase, the ICU had an average of 517 admissions, 71.7 CVC insertions, 1,146.3 central line days, and 8.3 CLABSI events. In the intervention phase, these averages declined to 417.5 admissions, 60.8 insertions, 709 central line days, and 4.5 CLABSI events. In the post-intervention phase, the averages further dropped to 435.7 admissions, 54.3 insertions, 422 central line days, and 3 CLABSI events.

### The difference in DUR and CLABSI rates among the study phases

**Figure 2:**
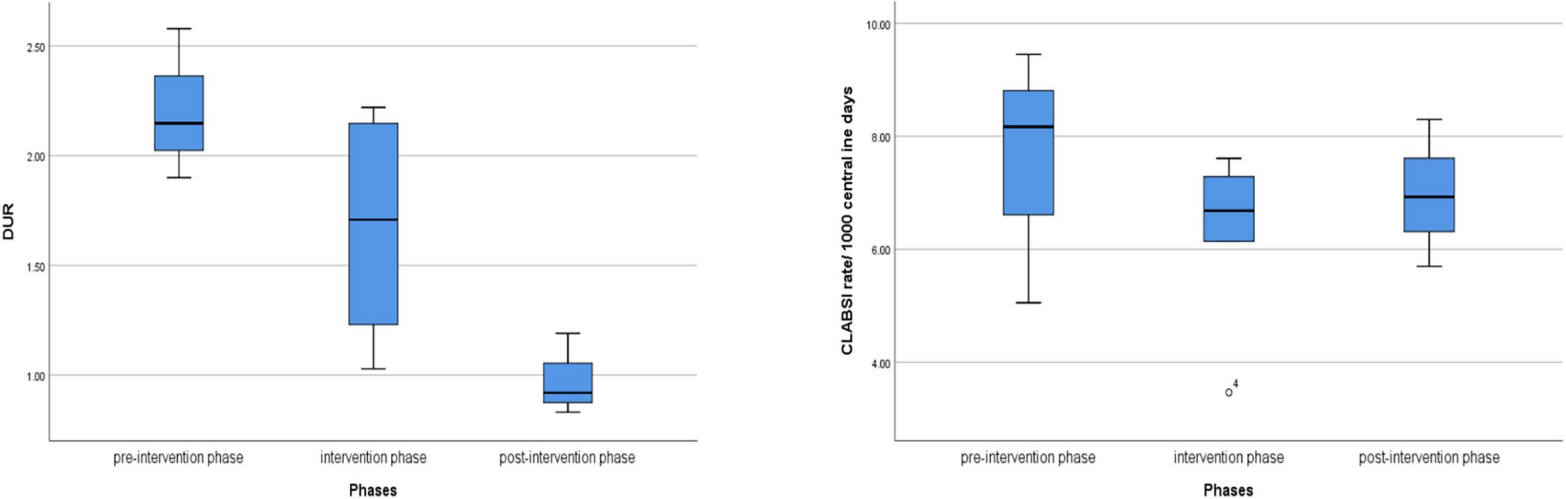
Box Plot chart for the median (IQR) of (CLABSI) rate per 1,000 CVC days (right) and DUR (left)among the pre-intervention, intervention & post-intervention phases. Box plots illustrate the median, interquartile range, and outliers for CLABSI rate (right) and Device Utilization Ratio (left) across the pre-intervention, intervention, and post-intervention phases. A downward trend in DUR is observed, with a less consistent pattern in CLABSI rate reduction. The sustained DUR decrease is evident in the post-intervention period.

The study demonstrated a statistically significant reduction in DUR during both the post-intervention and intervention phases when compared to the pre-intervention phase (0.98 < 1.67 < 2.21). Notably, the decrease in the DUR during post-intervention was significantly more than that observed during the intervention phase (54.35% reduction vs. 8.87% reduction, *p* = 0.02*). Regarding the CLABSI rate, it reduced from (7.56 to 6.97 per 1,000 CVC days), showing a non-statistically significant reduction (*p* = 0.6) as shown in Table 2 and Figure 2.

**Table 2:** Comparing CLABSI rate and DUR among the study phases:

Variables	CLABSI rate/1000 CVC days	95% CI	*p*-value	DUR	95% CI	*p*-value
Pre-intervention	7.56 ± 2.26 (5.05–9.45)	(1.94–13.17)	0.3^a^	2.21 ± 0.34 (1.90–2.58)	(1.36–3.1)	0.1^a^
Intervention	6.31 ± 1.49 (3.47–7.61)	(4.74–7.87)	0.6^b^	1.67 ± 0.52 (1.03–2.22)	(1.13–2.21)	0.006*^b^
Post-intervention	6.97 ± 1.31 (5.70–8.30)	(3.74–10.2)	0.5^c^	0.98 ± 0.19 (0.83–1.19)	(0.51–1.44)	0.04*^c^
Percent of reduction	30.5%^i^–13.4%^ii^			8.87%^i^–54.35%^ii^		
*p*-value#	0.57			0.02*		

Data were presented as mean ±SD (range), CI, Confidence Interval# *p*-value for independent samples Kruskal Wallis test. *Statistically significant (*p*-value≤0.05). ^a^pre-intervention vs. intervention, ^b^pre-intervention vs. post-intervention, ^c^intervention vspost-intervention. ^i^percent of reduction in phase II, ^ii^percent of reduction in phase III.

### The reduction trend of DUR and CLABSI rates

**Figure 3:**
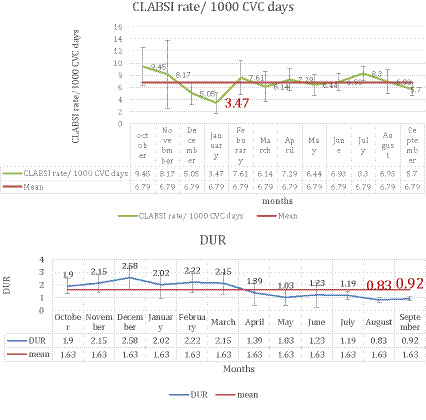
CLABSI rate—and DUR through the study phases. Charts show monthly CLABSI rate per 1,000 CVC days (top) and DUR (bottom) across all three study phases. A marked drop in CLABSI rates is evident in January (Highlighted) following intervention initiation, with rates remaining below baseline thereafter. DUR declined steadily, reaching its lowest levels in August and September (highlighted in red), indicating a temporal association with the implemented quality improvement measures.

As shown in Figure 3, the CLABSI rate per 1,000 CVC days was at its lowest in January, coinciding with the commencement of the intervention. Although there was an increase thereafter, the rate remained lower than that observed during the pre-intervention phase. Concerning DUR, the reduction trend continued throughout both the intervention and post-intervention phases, with the lowest DUR observed in August and September (0.83 and 0.91), indicating a sustained decrease following the intervention phase.

**Figure 4:**
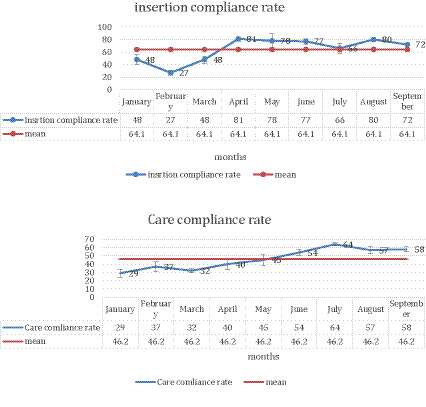
Insertion compliance (above)-maintenance compliance rates (below) through the intervention & post-intervention phases. Charts depict changes in compliance rates for insertion bundle (top) and maintenance bundle (bottom) during intervention and post-intervention phases. A modest increase in insertion bundle compliance (62.5 to 72%, *p* = 0.6) and a significant rise in maintenance bundle compliance (39.5 to 59.7%, *p* = 0.01), reflect greater improvement in routine CVC maintenance.

The change in the insertion and maintenance compliance rates during the intervention and post intervention phases: The insertion compliance rate improvement remained stable around the mean throughout both the intervention and post-intervention phases. Conversely, the maintenance compliance rate exhibited a positive trend, with more significant improvement noted in the post-intervention phase, as illustrated in Figure 4. The maintenance compliance rate demonstrated a statistically significant increase in the post-intervention phase compared to the intervention phase (59.7% vs. 39.5%, *p* = 0.01*), reflecting 51.1% improvement. However, the increase in insertion compliance was not statistically significant (72% vs. 62.5%, *p* = 0.6), indicating 21.4% improvement, as shown in Table 3.

**Table 3:** Insertion and maintenance compliance rates:

Variables	Insertion compliance rate	95% CI	*p*-value	Maintenance compliance rate	95% CI	*p*-value
Intervention	62.5 (42.7–78.7)	(36.7–82.9)	0.6	39.5 ± 9.1 (29.0–54.0)	(29.95–49.1)	0.01*
Post-intervention	72 (66–72)	(55.22–90.11)	21.4%	59.7 ± 3.8 (57–64)	(50.3–69.1)	51.1%

Data presented as mean± SD (range) or median (IQR). CI, Confidence Interval.

### The correlation between CLABSI rates with the insertion and maintenance bundles compliance

Table 4 illustrates a non-significant correlation between CLABSI rate and compliance rates. While in (Table 5), a detailed analysis of compliance rates for selected components within the maintenance bundle revealed that proper hand hygiene demonstrated a statistically significant strong negative correlation with the CLABSI rate in the post-intervention phase (*r* = −0.95, *p* = 0.02*).

**Table 4:** Correlation between CLABSI rate per 1,000 CVC days with insertion and maintenance compliance bundles during the intervention and post intervention phases.

Compliance bundles	CLABSI rate/1000 CVC days			
	Intervention phase		Post intervention phase	
	*r*	*p-*value	*r*	*p-*value
Insertion	−0.16	0.7	−0.45	0.7
Maintenance	−0.81	0.39	−0.56	0.24

*r* = correlation coefficient, *Statistically significant (*p*-value≤0.05).

**Table 5:** Correlation between CLABSIRate per 1,000 CVC days with the compliance rate of selected items in the maintenance bundle during intervention and post-intervention phases.

Variables	CLABSI rate/1000 CVC days			
	Intervention phase		Post intervention phase	
	*R*	*p-*value	*R*	*p-*value
Need for CVC for patients	−0.35	0.4	−0.83	0.4
Proper hand hygiene	−0.65	0.15	−0.95	0.02*
Alcohol Used appropriately	−0.24	0.64	−0.83	0.4

*r* = correlation coefficient, *Statistically significant (*p*-value≤0.05).

## Discussion

In the intensive care unit under investigation, the designed quality improvement initiative demonstrated that targeted, context-specific interventions can significantly improve CVC care and reduce CLABSI rates, even in resource-constrained intensive care settings. Comprehensive analysis of local risk factors, including prolonged catheterization, multi-lumen devices, and limited resources, guided the implementation of standardized protocols, staff training, and regular audits. These efforts resulted in a marked reduction in CLABSI incidence, a significant decline in DUR, and improved compliance with insertion and maintenance bundles. Enhanced adherence to hand hygiene and routine CVC necessity assessments further contributed to improved outcomes, underscoring the value of sustained behavioral and process changes in achieving safer and more sustainable critical care practices.

Prior to the initiation of the current QI initiative, CLABSI were reported in 56% of patients with inserted CVCs. Although this was not a precisely calculated rate due to the lack of a standardized definition, it was sufficiently high to prompt the current initiative and highlight the necessity for effective intervention. This percentage was slightly higher than that reported by Mathur et al., who found that 45.7% of bloodstream infections were CLABSIs (23). It also exceeds the findings of an earlier study conducted in Egypt, which identified a CLABSI rate of 34.19% in their intensive care unit (24), and another study conducted in the same unit years earlier (7). As published earlier (25, 26), we monitored outcomes using the CLABSI rate, dependent on positive cultures and institutional norms for culture ordering, and DUR, independent of lab testing.

During the preintervention phase, an accurate calculation revealed a CLABSI rate of 7.56 ± 2.26 per 1,000 CVC days This rate was marginally higher than those reported in several studies (5, 23, 24), and significantly higher than the rate reported in a study conducted at a private accredited hospital in Egypt (27). The highest CLABSI rate observed in the later study was 4.3 per 1,000 CVC-days (95% CI 3.7–5.0) in medical ICUs, while the rate in surgical ICUs was 3.5 per 1,000 CVC-days (95% CI 3.2–3.7). This discrepancy can be attributed to the rigorous infection control program and bundle application enforced according to joint commission standards at that hospital (27). Variations in reported CLABSI rates may stem from the differences in ICU population characteristics, healthcare workers’ awareness of CVC risks, and compliance with infection control policies by staff and patients (9, 11, 27–30).

Evidence-based insertion and maintenance bundles have been shown to significantly reduce CLABSI rates in hospital settings (11, 31–34). One PDSA cycle in our study used checklists to monitor insertion and maintenance, while the next focused on staff education for proper CVC insertion and maintenance procedures. The designed intervention resulted in a non-statistically significant reduction in CLABSI rates per 1,000 CVC days No line lock solutions were used during this intervention, which may have contributed to the non-significant change in CLABSI rates, as the initiative primarily targeted procedural and maintenance compliance rather than intraluminal biofilm prevention. Although the reduction did not reach statistical significance, the downward trend remains clinically meaningful within the context of a small sample size and limited study duration. The improvement most likely reflects enhanced process reliability rather than a definitive outcome difference.

Similar findings were reported by Odada et al. (35) and Chandramohan et al. (36); their results also indicated non-significant reductions in CLABSI rates. Factors such as staffing issues, workload, resource constraints, and patient acuity likely influenced outcomes in all these studies (35–37). In contrast, Latif and his colleagues conducted a long-term, complex intervention in Pakistan over 9 years and achieved a significant reduction (77%) in the NICU, with an overall 36% reduction in CLABSI rates across all ICUs included in their study. A critical factor contributing to this significant change was the long-term sustainability and continuity of their intervention program, transforming the initiative from a practice modification to actual system and behavioral change (38).

The apparent reduction in our study, though statistically non-significant, suggest that context-adapted implementation and engagement strategies can produce measurable gains in safety performance. Nonetheless, the results should be interpreted cautiously, and larger multicenter studies are warranted to confirm the generalizability and long-term impact of such interventions.

Several implementation factors may explain the observed improvement. Structured, hands-on training increased technical proficiency and awareness among staff, while the multidisciplinary CLABSI working group fostered engagement, accountability, and shared ownership of infection prevention goals. The frequency and immediacy of audit-feedback cycles were also critical—daily observations and real-time corrective feedback helped sustain compliance and behavioral change. Leadership support further reinforced adherence to protocols despite workload pressures.

These findings suggest that continuous monitoring, staff empowerment, and iterative learning cycles may support inferred long-term improvements.

Our findings align with global evidence demonstrating the effectiveness of QI interventions and central line care bundles in reducing CLABSI rates, even within resource-constrained ICUs. A comprehensive meta-analysis by Blot et al. (39) reported that implementation of central line bundles reduced CLABSI rates by approximately 56% (RR 0.44, 95% CI 0.39–0.50), with consistent benefits across diverse hospital settings. Similarly, the multicenter survey by Alp et al. (40) and an international multicenter analysis by Devrim et al. (28) confirmed widespread adoption of CLABSI prevention bundles in LMICs but noted that full compliance remains uncommon due to staffing shortages, inconsistent training, and variable surveillance quality. Our study’s downward trend in CLABSI rates, although not statistically significant, how contextual implementation strategies supported by leadership and continuous audit-feedback mechanisms may contribute to inferred sustained improvements. Furthermore, the multicenter QI project by Samanta et al. (41) demonstrated a 43% reduction in CLABSI rates (from 14.1 to 8.0 per 1,000 central line-days, *p* = 0.04) through multidisciplinary teamwork, staff empowerment, and real-time monitoring factors that closely parallel our own intervention design.

From a regional benchmarking perspective, our baseline rate (7.56 per 1,000 CVC-days) was comparable to pooled estimates from INICC, which reported 8–12 CLABSIs per 1,000 device-days in LMIC ICUs—five times higher than rates in high-income countries (5). Furthermore, a comprehensive meta-analysis of 59 prospective studies demonstrated that the central line bundle reduced CLABSI by approximately 56% (RR 0.44, 95% CI 0.39–0.50) across diverse settings, though substantial heterogeneity was noted due to variations in implementation fidelity and study design. Even among high-quality studies, the reduction remained significant (52, 95% CI 32–66%), underscoring that consistent bundle application yields meaningful clinical impact (42). A recent Chinese meta-analysis (2008–2023) further supported this disparity, reporting an overall weighted CLABSI rate of 2.65 per 1,000 catheter-days (95% CI: 2.57–2.73), with the highest rates observed in adult ICUs in northern China (5.13 per 1,000, 95% CI: 4.23–6.02) (43). Although these rates are lower than those seen in some LMICs studies, they still exceed CDC-NHSN benchmarks, underscoring the global need for enhanced surveillance standardization and stronger infection prevention systems. In Egypt, national surveillance by El-Kholy et al. documented higher CLABSI rates (≈9.1 per 1,000 line-days) (6) Together, these data confirm that CLABSI remains a major preventable source of morbidity worldwide and that bundle-based QI initiatives, when implemented with fidelity and sustained engagement, have the potential to yield clinically meaningful improvements with inferred environmentally sustainable benefits in resource-limited settings, even if statistical significance is not always achieved in individual studies.

In all instances, monitoring the change in rates over time by a run chart helps healthcare facilities visualize trends in CLABSI rates, identify potential issues, and assess the effectiveness of interventions. Our results demonstrated a significant reduction in DUR during the intervention and post-intervention phases compared to the pre-intervention phase, with the largest reduction being 54.35% (*p* = 0.02) in the post-intervention phase. This demonstrated a greater reduction in DUR than those reported by Chandramuhan et al., who noted a decrease from 46% (pre-intervention) to 37% (post-intervention) (36). These findings reflected a successful focused strategy that targeted the reduction of unnecessary central lines and that was implemented as part of the intervention phase. This included daily assessments of central line necessity during clinical rounds using a standardized checklist, prompt documentation of line indications, and active reinforcement of early CVC removal when no longer clinically indicated. ICU staff were trained to prioritize line necessity in decision-making, and visual reminders were used to support daily evaluation. These efforts were monitored by the CLABSI working group and reinforced through feedback during multidisciplinary meetings.

We observed a significant improvement in compliance with maintenance bundles (*p*-value = 0.01*), indicating a 51.1% improvement overall, and a 21.4% improvement in insertion compliance (*p*-value = 0.6). Previous studies have also reported increased bundle compliance following interventions (32–35). The most effective parameters from these studies, which our study did not incorporate, include electronic monitoring (35) and extended study duration (33, 44). Furuya et al. reported a 33% reduction in CLABSI rates with over 75% compliance to all bundle components. Compliance below 75%, or to only one or two components, did not significantly improve infection rates (45). Lee et al., concluded that perfect application of all bundle components is crucial for preventing CLABSIs (46).

As part of our training needs assessment, we evaluated performance on each bundle component. We calculated the correlation between individual elements, and CLABSI rate. Exploratory analysis suggested a statistically significant strong negative correlation of hand hygiene compliance before CVC maintenance with the CLABSI rate in the post-intervention, although these findings should be interpreted cautiously given statistical limitations. Comparable findings were reported earlier, Mohapatra et al. found a significant improvement in hand hygiene compliance in the post-intervention phase, with a 48% reduction in CLABSI rates compared to the pre-intervention phase (47).

A key strength of this study is its structured, multi-phase design using repeated PDSA cycles tailored to a high-risk ICU environment in a resource-limited setting, with explicit mapping of bundle components to established international guidelines (CDC, SHEA/IDSA, WHO) to enhance scientific validity, comparability, and global relevance. Active engagement of frontline staff, direct observation, and real-time feedback enabled context-specific interventions that improved care processes. The inclusion of exploratory correlation analysis between bundle adherence and clinical outcomes provided additional insight into the drivers of change and informed targeted improvement efforts. Captivatingly, the observed increase in compliance rates strongly motivated ICU leadership to support continued implementation and may support inferred sustainability outcomes. Beyond infection prevention outcomes, this initiative also suggests a potential positive environmental impact. The significant reduction in the device utilization ratio reflects decreased use of central venous catheters and associated single-use consumables such as sterile drapes, syringes, and dressing kits. These reductions imply potential environmental benefits, as decreased the generation of medical waste and the consumption of disposable plastics, although they were not directly quantified. Embedding sustainability within infection control practices supports the WHO 2022 global call for “greener healthcare,” particularly relevant for LMICs where waste management systems are often underdeveloped. Integrating environmental awareness into staff training and daily audits may foster a dual culture of safety and resource stewardship, representing inferred cost-effective sustainable infection prevention.

## Limitations

This study’s generalizability may be limited by its single-center scope, exclusion criteria, short post-intervention follow-up, and relatively small number of CLABSI events, which reduce statistical power. Given the limited sample size, adjustment for potential confounders such as patient acuity, and central line days was not feasible; however, these descriptive data provide important context for interpreting observed improvements. Future studies with larger samples, longer follow-up, and stratified analyses are needed to control potential confounders and strengthen the generalizability of findings.

While this study provides preliminary evidence linking improved infection control with inferred environmental sustainability, quantitative measures of waste reduction (e.g., volume or weight of disposables avoided) were not recorded. Future studies should include environmental metrics such as waste audits or carbon footprint assessments to better quantify the ecological benefits of infection prevention initiatives.

## Conclusion

This quality improvement initiative demonstrates that structured, low-cost, and context-sensitive interventions can enhance central venous catheter safety while potentially supporting environmental sustainability in resource-constrained ICUs. By reducing unnecessary catheter use and reinforcing evidence-based maintenance practices, the project achieved both clinical and ecological benefits—improving compliance and decreasing disposable material consumption. The absence of line lock solutions, while reflecting local resource constraints, may have limited further reductions in CLABSI rates, underscoring the need to explore their role in future interventions.

## Recommendations

Incorporating sustainability indicators—such as waste reduction, material reuse potential, and energy-efficient practices—into quality improvement frameworks can further promote *green critical care* and potentially environmentally responsible infection prevention. Hospitals should also integrate environmental stewardship into infection control training, emphasizing that patient safety and planetary health are mutually reinforcing goals. Additionally, digital surveillance tools and continuous education modules could enhance real-time monitoring and scalability across healthcare systems.

## Data Availability

The datasets generated and/or analyzed during the current study are available from the corresponding author upon reasonable request.
