# Supplementary material for: Central venous catheter care and the healthcare–environment interface: outcomes of a quality improvement initiative in a resource-constrained ICU
Source: Front Public Health. 2025 Dec 12;13:1713370. doi: 10.3389/fpubh.2025.1713370 (PMC12741089; doi:10.3389/fpubh.2025.1713370)
Supplement: Supplementary file 1 [file Data_Sheet_1.pdf]

Revised Standards for Quality Improvement Reporting Excellence

(SQUIRE 2.0) September 15, 2015

Text Section and Item Name	In manuscript
Title and Abstract	
1. Title	Central Venous Catheter Care and the Healthcare–Environment Interface: Outcomes of a Quality Improvement Initiative in a Resource-Constrained ICU
2. Abstract	**Background:** Central line-associated bloodstream infections (CLABSI) are major indicators of healthcare quality and patient safety, particularly in resource-constrained intensive care units (ICUs). **Aim:** This study aimed to evaluate a pre-post quality improvement (QI) initiative designed to optimize central venous catheter (CVC) insertion and maintenance, reducing CLABSI rates, and promoting more sustainable critical care practices in a university hospital ICU. **Methodology:** A one year pre–post QI study (October 2022- September 2023) was conducted in an emergency ICU of a university hospital in Egypt hospital. The study was structured according to the SQUIRE 2.0 framework and using sequential Plan-Do-Study-Act (PDSA) cycles. All ICU patients who required CVCs in the investigated ICU and met inclusion criteria were included. The intervention included: 1) standardizing CLABSI definitions and rate calculations, 2) introducing CVC insertion and maintenance checklists with daily audits, 3) targeted staff education and training, and 4) forming a multidisciplinary CLABSI working group for monitoring and feedback. Over six months, process improvements were implemented, followed by three months of impact measurement, including assessing CVC insertion/maintenance compliance, CLABSI rates, and central line utilization ratio (DUR), with all data collected manually. **Results:** A total of 1,370 patients and 2,277 CVC days were observed. The CLABSI rate declined from 7.56 ± 2.26 to 6.97 ± 1.31 per 1,000 CVC days (−13.4%), and the device utilization ratio (DUR) decreased significantly from 2.21 ± 0.34 to 0.98 ± 0.19 (p = 0.02). Maintenance bundle compliance improved from 39.5% to 59.7% (p=0.01), and insertion compliance increased from 62.5% to 72% (p=0.6). Hand hygiene adherence demonstrated a strong negative correlation with CLABSI rates (r=-1.0, p=0.02). **Conclusion:** This single-center study had a small sample size and short post-intervention follow-up, which may limit generalizability. Nonetheless, the initiative demonstrates that structured, low-cost QI interventions can improve compliance, reduce device use, and support safer, more sustainable ICU care. Continuous monitoring and multicenter validation that integrate infection control with environmental stewardship are essential to sustain improvements and minimize the environmental footprint of critical care.
Introduction	**Why did you start?**
3.Problem Description	Introduction:<br>CVC use increases the risk of potentially fatal bloodstream infections (2). Many health care institutions have set targets to minimize central line-associated bloodstream infections (CLABSIs) to enhance patient outcomes and lower healthcare expenses (3).<br>Approximately 250,000 bloodstream infections occur annually in U.S. hospitals, with nearly 80,000 CLABSIs reported in ICUs each year (4). The

	International Nosocomial Infection Control Consortium (INICC) estimates a pooled incidence of 4.1 per 1,000 central line-days over five years (5). Middle-income countries (MICs) report considerably higher CLABSI rates than high-income countries, likely reflecting disparities in infrastructure, resources, and adherence to prevention protocols. In Egypt, for example, a multicenter study across three ICUs reported an average CLABSI rate of 9.1 per 1,000 central line-days (6).
4.Available knowledge	Introduction<br>The International Nosocomial Infection Control Consortium (INICC) estimates a pooled incidence of 4.1 per 1,000 central line-days over five years ([5). Middle-income countries (MICs) report considerably higher CLABSI rates than high-income countries, likely reflecting disparities in infrastructure, resources, and adherence to prevention protocols. In Egypt, for example, a multicenter study across three ICUs reported an average CLABSI rate of 9.1 per 1,000 central line-days (6).
5. Rationale	Methods:<br>The Quality Improvement (QI) approach was chosen for its capacity to systematically identify gaps in care, implement targeted, evidence-based intervention, and facilitate ongoing monitoring through iterative cycles. This methodology enables timely adjustments, fosters staff involvement, and promotes adherence to best practices within high-risk and dynamic settings.
6. Specific aims	This study aimed to evaluate a pre-post quality improvement (QI) initiative designed to optimize central venous catheter (CVC) insertion and maintenance, reducing CLABSI rates, and promoting more sustainable critical care practices in a university hospital ICU.
Methods	What did you do?
7. Context	**Methods:**<br>This initiative was conducted in the emergency ICU of a university hospital in Egypt, a resource-constrained setting with a high burden of CLABSIs. Before the intervention, the unit lacked standardized CLABSI definitions, updated CVC care policies, and structured staff training. Frequent turnover of junior staff, absence of insertion qualifications, and limited infection control oversight further contributed to infection risk. These contextual factors shaped the design and implementation of the interventions and highlighted the necessity of tailoring improvement strategies to local conditions.
8. Intervention(s)	8.a<br>The intervention phase spanned six months and involved four Plan-Do-Study-Act (PDSA) cycles (Figure 1). Interventions were based on a prior unpublished QI study and refined iteratively during implementation.<br><br>8.b<br>It was carried out by a multidisciplinary team, with specific roles assigned to nurses, physicians, and infection control professionals.
9. Study of the Intervention(s)	The study assessed the intervention's impact using pre-, during-, and post-intervention comparisons over a one-year period (October 2022 – September 2023). Baseline data and stakeholder input identified key gaps in CVC insertion and maintenance. Process and outcome indicators were monitored throughout to evaluate changes attributable to the interventions. Trends and correlations were explored to understand intervention effects and their sustainability.
10. Measures	Measures subheadings in the methods section
11. Analysis	11a.

	Descriptive statistics were used to summarize patient characteristics and outcome variables across the three phases. Differences in means were analyzed using one-way ANOVA, and categorical data were compared using the Chi-square test. Statistical significance was set at $p < 0.05$.<br>11b.<br>Variation over time was assessed across the pre-, intervention-, and post-intervention phases. Data for admissions, CVC insertions, central line-days, and CLABSI events were compared using ANOVA and Chi-square tests. Correlation analysis explored time-dependent associations between bundle adherence and infection reduction.
12. Ethical Considerations	This study was conducted in accordance with the Declaration of Helsinki. Ethical approval was obtained from the institutional review board [No: ZU-IRB#10902-19/7-2023]. Written informed consent for participation in this study was provided by the participants' legal guardians/next of kin.
Results	**What did you find?**
13. Results	a. Initial steps of the intervention(s) and their evolution over time:<br>The project was implemented in three sequential phases—pre-intervention, intervention, and post-intervention—enrolling 1370 patients aged 19–78 years (≈ 60 % male, 40 % female). Across all phases, participants had comparable demographic and clinical profiles, including Glasgow Coma Scale, APACHE II, and SOFA scores, with no statistically significant differences. During the intervention, central venous catheter (CVC) insertion and maintenance bundles were introduced and progressively refined through ongoing staff training, audit-feedback, and reinforcement during daily rounds. Continuous improvement cycles led to sustained practice changes in the post-intervention phase.<br>b. Process and outcome results:<br>A clear downward trend was observed in both device utilization and CLABSI rates over time.<br>• Pre-intervention: 2.21 DUR; 7.56 CLABSI / 1,000 CVC days<br>• Intervention: 1.67 DUR; 6.31 CLABSI / 1,000 CVC days<br>• Post-intervention: 0.98 DUR; 6.97 CLABSI / 1,000 CVC days<br>The DUR reduction was statistically significant between the pre-intervention and post-intervention phases (54.35 % reduction, $p = 0.02$) and also significant between intervention and post-intervention ($p = 0.04$). The CLABSI rate declined by 30.5 % overall (7.56 → 6.97 per 1,000 CVC days) but did not reach statistical significance ($p = 0.6$). Insertion-bundle compliance improved from 62.5 % to 72 % (+21.4 %, $p = 0.6$), while maintenance-bundle compliance rose significantly from 39.5 % to 59.7 % (+51.1 %, $p = 0.01$). Figure 2 and Table 2 illustrate a consistent and sustained decline in DUR with a less pronounced yet clinically relevant reduction in CLABSI rates.<br>c. Contextual elements that interacted with the intervention(s):<br>Outcomes were shaped by the ICU's high-acuity surgical case mix and resource constraints. Leadership commitment, infection-control oversight, and multidisciplinary teamwork facilitated adoption of the bundles. Frequent staff rotation initially limited compliance but was mitigated by structured onboarding and visual reminders. The visible decline in CLABSI rates reinforced staff motivation and adherence.<br>d. Observed associations between outcomes, interventions, and contextual elements<br>Improved bundle compliance correlated with reduced infection risk. While overall CLABSI reduction was non-significant, hand-hygiene compliance within the maintenance bundle showed a strong negative correlation with

	CLABSI rate (r = –0.95, p = 0.02) during the post-intervention phase, highlighting its critical role in sustained infection prevention.<br>e. Unintended consequences:<br>No adverse outcomes or unexpected complications occurred. Early in the intervention, audit and training activities increased workload; however, workflow integration improved as staff became familiar with the bundle processes. The minor opportunity costs were offset by improvements in patient safety indicators.<br>f. Missing data:<br>Data completeness exceeded 95 % across all measures. Occasional missing entries during staff transitions were verified through infection-control logs and excluded from denominator calculations, minimizing bias and preserving analytic validity.
Discussion	Discussion
14. Summary	a-the key findings in the study: declined CLABSI rate, the device utilization ratio (DUR) decreased significantly, improvement of maintenance and insertion bundle compliance. These findings were relevant to aims of current QI study<br>b. Particular strengths of the project<br>A key strength of this study is its structured, multi-phase design using repeated PDSA cycles tailored to a high-risk ICU environment in a resource-limited setting. Active engagement of frontline staff, direct observation, and real-time feedback enabled context-specific interventions that improved care processes
15. Interpretation	**a. Nature of the association between the intervention(s) and the outcomes:**<br>The multi-phase intervention demonstrated a clear temporal association between the implementation of evidence-based central line bundles and improvements in clinical performance indicators. The significant decline in device utilization ratio (DUR) and the non-significant but clinically relevant reduction in CLABSI rates coincided with progressive improvement in bundle adherence, particularly in the maintenance phase. The strong negative correlation between hand hygiene compliance and CLABSI rate (r = –0.95, p = 0.02) further supports the causal link between consistent application of key preventive practices and infection reduction. These findings suggest that interventions through reinforcing staff knowledge, standardizing procedures, and continuous feedback—contributed directly to safer central line care and sustained process improvement within the ICU.<br>**b. Comparison of results with findings from other publications**<br>The observed decline in CLABSI rates and device utilization aligns with trends reported in both national and international quality improvement (QI) studies. Similar initiatives applying bundle-based interventions and PDSA cycles in resource-limited ICUs have achieved 25–60 % reductions in CLABSI incidence, particularly when staff engagement and audit-feedback were emphasized. Although the reduction in CLABSI in this study did not reach statistical significance, the direction and magnitude of change were consistent with published outcomes from multicenter QI projects in middle-income countries. These parallels indicate that structured, low-cost, and context-sensitive interventions can yield meaningful improvements even in constrained environments, reinforcing the generalizability and relevance of the current findings.<br>**c. Impact of the project on people and systems:**<br>The intervention had a positive and measurable impact on both staff compliance and the ICU's infection prevention system. Continuous feedback, visible infection rate monitoring, and direct leadership

	involvement fostered a stronger culture of accountability and teamwork. Nursing and medical staff demonstrated greater adherence to infection control measures during insertion and maintenance. The observed improvement in maintenance compliance and sustained reduction in device utilization reflected not only procedural changes but also enhanced situational awareness and ownership of patient safety practices. At the system level, the project established a structured infection surveillance and audit mechanism that remained active beyond the study period, supporting long-term sustainability.<br>**d. Reasons for any differences between observed and anticipated outcomes, including the influence of context:**<br>While the CLABSI rate showed a downward trend, the reduction did not reach statistical significance, likely due to the limited study duration, small number of CLABSI events. Inherent variability in patient acuity and line-days, though were not analyzed, may have an impact. High staff turnover and the resource-constrained ICU setting may have delayed full implementation fidelity. Despite these contextual limitations, the consistent improvement in process measures (especially maintenance bundle adherence and DUR reduction) indicates that behavioral and system-level changes were successfully achieved, even if outcome stabilization required a longer follow-up period.<br>**e. Costs and strategic trade-offs, including opportunity costs:**<br>Implementing the intervention required dedicated time for staff training, observation, and audit-feedback sessions, temporarily increasing workload and consuming additional sterile supplies. These short-term resource investments represented modest opportunity costs as personnel and materials were diverted from routine duties. However, these costs were offset by the potential reduction in infection-related morbidity, antibiotic use, and hospitalization duration, highlighting a favorable trade-off between short-term resource utilization and long-term patient safety gains. The initiative also introduced more efficient use of CVCs through necessity assessment, contributing to cost containment and environmental sustainability.
16. Limitations	**a. Limits to the generalizability of the work:**<br>The generalizability of this study is limited by its single-center design, which reflects the characteristics and practices of one surgical emergency ICU within a university hospital. The short post-intervention follow-up period and the relatively small number of CLABSI events reduced statistical power and may limit extrapolation of results to other settings. Additionally, the study's focus on a specific clinical environment and resource-constrained context may restrict its direct applicability to ICUs with different patient populations or resource profiles.<br>**b. Factors that might have limited internal validity:**<br>Potential threats to internal validity include the absence of adjustment for confounding variables such as patient acuity, central line-days variation, and other unit-level factors that could influence infection rates. The non-randomized, pre–post design introduces the possibility of temporal confounding or bias from concurrent improvements in practice unrelated to the intervention.<br>**c. Efforts made to minimize and adjust for limitations:**<br>Descriptive analysis of clinical and contextual variables was conducted to ensure comparability across study phases, providing a transparent basis for interpreting observed improvements. Rigorous audit-feedback and data validation processes minimized measurement bias. While statistical adjustment for confounders was not feasible due to sample size constraints,

	consistency in ICU protocols and staff training helped reduce variation across phases.<br>The study also provides preliminary evidence linking infection prevention with environmental sustainability, although quantitative measures of waste reduction (e.g., disposable volume or carbon footprint) were not recorded. Future multicenter studies with larger samples, extended follow-up, and inclusion of environmental performance indicators (such as waste audits or resource utilization metrics) are warranted to strengthen causal inference and generalizability.
17. Conclusions	**a. Usefulness of the work:**<br>This quality improvement initiative demonstrates that structured, low-cost, and context-sensitive interventions can substantially improve central venous catheter safety in a resource-constrained surgical ICU. The project confirmed that systematic implementation of evidence-based insertion and maintenance bundles can lead to measurable improvements in patient safety, care consistency, and clinical outcomes.<br>**b. Sustainability:**<br>Sustainability was achieved through continuous staff engagement, routine audits, and leadership support, allowing adherence to remain stable beyond the active intervention phase. The project's integration of environmentally conscious practices, such as minimizing unnecessary catheter use and optimizing resource consumption, further enhanced its long-term viability by aligning safety and sustainability goals.<br>**c. Potential for spread to other contexts:**<br>The approach and outcomes of this initiative are adaptable to other ICUs, particularly in low- and middle-income countries where similar resource and staffing challenges exist. Its stepwise, data-driven framework—emphasizing education, compliance monitoring, and iterative feedback—provides a practical model for replication in comparable healthcare environments.<br>**d. Implications for practice and further study in the field:**<br>The findings highlight the value of embedding environmental stewardship principles within infection prevention programs. Hospitals can integrate sustainability indicators—such as waste reduction, material reuse, and energy-efficient practices—into quality improvement frameworks to promote green critical care. Incorporating these dual objectives supports both patient safety and ecological responsibility.<br>**e. Suggested next steps:**<br>Future work should focus on multicenter collaborations, longer follow-up durations, and the inclusion of quantitative sustainability metrics (e.g., waste audits, carbon footprint assessments) to more comprehensively measure the ecological benefits of infection control initiatives. Additionally, digital surveillance tools and continuous education modules could enhance real-time monitoring and scalability across healthcare systems.
Other information	
18. Funding	No sources of funding
